# Methemoglobinemia Secondary to a Traditional Healing Practice Using Mothballs: A Need of Pediatric Vigilance

**DOI:** 10.7759/cureus.41192

**Published:** 2023-06-30

**Authors:** Varun Anand, Dilip K Venkatesan, Pugazhenthan T, Md. Naseem, Santosh K Rathia

**Affiliations:** 1 Trauma and Emergency/Pediatric Emergency Medicine, All India Institute of Medical Sciences Raipur, Raipur, IND; 2 Pediatric Emergency Medicine, All India Institute of Medical Sciences Raipur, Raipur, IND; 3 Pharmacology and Therapeutics, All India Institute of Medical Sciences Raipur, Raipur, IND

**Keywords:** traditional healing practice, pediatric emergency medicine, emergency department, exchange transfusion, naphthalene, moth ball, methemoglobinemia

## Abstract

Acute-onset unexplained hypoxemia persisting despite 100% oxygen has a limited differential diagnosis but poses a challenging diagnostic dilemma. Methemoglobinemia, a hemolytic condition, may lead to significant complications if it goes undiagnosed during the critical golden hour of an emergency department (ED) presentation. This case report presents the clinical details of a 30-month-old child with acute intravascular hemolysis evident by severe pallor and hemoglobinuria and severe hypoxia documented on pulse oximetry. During the ABCDE (Airway, Breathing, Circulation, Disability, Exposure) of the primary survey, "exposure" revealed the parent’s deliberate fastening of a mothball around the waist of the baby on the advice of a traditional healer, which was identified as the source of naphthalene toxicity. The swift intervention was undertaken for hypoxic respiratory compromise with 100% oxygen just after triage, and the naphthalene ball with the tied cloth was removed. Arterial blood gas and co-oximetry analysis confirmed the diagnosis of methemoglobinemia, and other laboratory tests suggested severe hemolytic anaemia as well as hemoglobinuria favouring intravascular hemolysis. With the exclusion of other common differentials for hemolytic anaemia, including sickle cell crisis, autoimmune hemolytic anaemia, hemolytic uremic syndrome, and G6PD deficiency, naphthalene exposure was considered the culprit for both hemolysis and methemoglobinemia. After obtaining the history of another similar episode of anaemia six months ago requiring blood transfusion, we retrospected on similar mothball exposure, but parents denied that, saying they were using the mothball only for the last 10 days on the advice of a local healer with intent to get rid of some evil power and sickness in their child. After analyzing the old records of prior hospitalization and getting assured of a normal report of G6PD level, intravenous methylene blue was administered. But in view of an inadequate response, a single blood volume exchange transfusion was performed during the ED stay only, which resulted in a notable reduction in subsequent methemoglobin levels and an improvement of the child's clinical condition by the second day. The child was discharged by the third day with no distress and no further episodes of hemoglobinuria, with detailed parental counselling and follow-up advice.

This case underscores the imperative need for timely recognition and effective management of methemoglobinemia in the paediatric population while emphasizing the potential hazards associated with naphthalene exposure. Further comprehensive investigations are warranted to elucidate optimal treatment strategies and explore long-term outcomes in similar clinical scenarios.

## Introduction

Acquired methemoglobinemia secondary to naphthalene toxicity is an acknowledged entity in the literature [1]. Naphthalene balls, commonly used as moth repellents in Indian households, pose a risk of exposure through ingestion, inhalation, and even dermal contact, leading to absorption and subsequent toxic effects. Even minimal amounts of absorption can have severe and potentially fatal consequences, including hepatic and renal dysfunction. Literature suggests there are various forms of this household or industrial chemical and many routes of naphthalene poisoning. Mothballs contain certain oxidative chemicals like naphthalene, and its by-product (alpha-naphthol) may cause oxygen-free radical-mediated cellular and subcellular injuries, including red blood cell hemolysis and methemoglobinemia. Although in India, various forms of mothballs are also available with different ingredients like naphthalene, paradichlorobenzene, and camphor, people use it mostly as a moth or insect repellent and a common repellent of bad odour from stored clothes, but in some regions, maybe with traditional thinking of old camphor composition or not knowing harms of currently used chemicals in mothballs, many people themselves or with the advice of local healers use it differently, like directly applying it to skin with coconut or other oil or tying it on some parts of the body.

In this case report, we present the clinical details of a 30-month-old male child who was brought to the emergency department (ED) with acute oxidative hemolysis as well as co-exiting methemoglobinemia, most likely both stemming from the unconventional practice of tying a mothball around his waist for the previous 10 days. It was based on a traditional practice of spiritual healing prevalent in the locality of the patient, where repeated physical sickness was thought to be the effect of some evil power.

## Case presentation

A 30-month-old male child presented to our paediatric ED on the morning of November 24, 2022, exhibiting signs of irritability, paleness, and rapid breathing, which warranted categorization as an "unstable-life-threatening condition" during the initial Paediatric Assessment Triangle (PAT) evaluation. As a response to red category triage, the emergency response system (ERS) of the ED was activated, and a multiparameter monitor along with 100% supplemental oxygen via a non-rebreathing mask (NRBM) was promptly attached to the child.

Primary assessment

**Figure 1:**
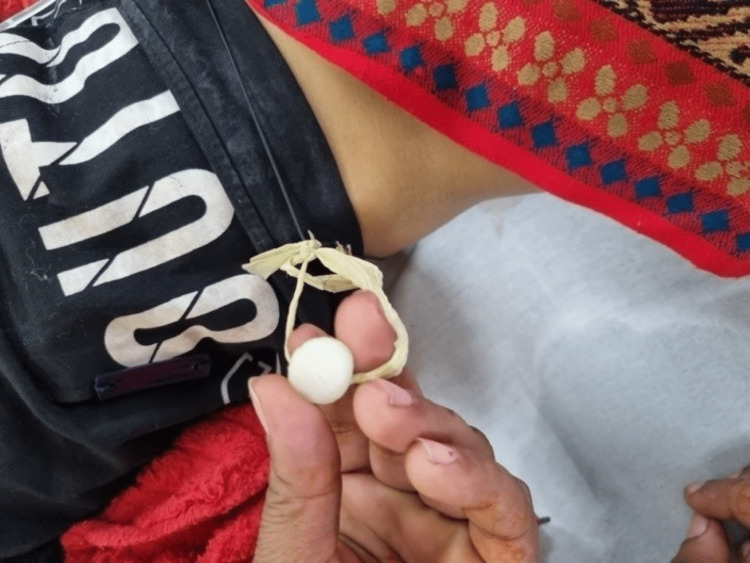
A mothball tied with a black cloth around the child's waist (for a traditional healing purpose)

The child's primary survey (ABCDE (Airway, Breathing, Circulation, Disability, Exposure) evaluation) revealed an open and maintainable airway (A), while the evaluation of breathing (B) showed tachypnoea with a respiratory rate of 52/min, normal breathing efforts without any visible retractions, bilateral equal air entry and symmetric chest rise without added adventitious sounds, and oxygen saturation of 68% on NRBM even at 15 L/min. In terms of circulation (C), the child exhibited a heart rate of 168 beats per minute with sinus rhythm on the ECG monitor; pallor was there, but peripheries were warm, with good central and peripheral pulses, a normal capillary refill time of 2-3 seconds, and blood pressure was normal (90/40 mmHg). During the evaluation of disability (D) part, the child displayed normal cerebral and brainstem functions, as well as a normal blood dextrose level. However, the child exhibited irritability on the spectrum of mild cortical dysfunction, which might be secondary to hypoxia. During the exposure (E) part of the quick evaluation, the child appeared pale and icteric, and surprisingly, a mothball (naphthalene) wrapped around his waist (as shown in Figure 1) was noticed by the team leader for case management.

Secondary assessment (focused history and examination)

A SAMPLE history (signs and symptoms, Allergies, Medications, Past medical history, Last meal, Events leading to the current problem) was obtained as a part of a secondary survey as per the standard paediatric ED case management approach, which revealed the presenting signs and symptoms of a sudden onset of brown-coloured urine and vomiting for two days, along with a one-day history of low-grade fever and jaundice. There was no recent drug intake, cough or cold, respiratory distress, cyanosis, bleeding from other sites, abdominal distension, decreased urine output, oedema, or recent blood transfusion. As the patient was from an endemic zone and a typically known at-risk community, a history of sickle cell disease (SCD) along with the possibility of any other bleeding or hemolytic disorder in the family were asked about to rule out. The child had no known allergies and was not taking any other offending medications. A guided history taking discovered that moth balls had been frequently used by the family for hygienic clothing storage as well as some ritual practice for spiritual healing for approximately six to seven months. As per his mother, they used these naphthalene balls as a repellent for insects or bad odour during winters and rainy seasons with stored clothes, and thinking that it was a safe household substance, they often used the clothes directly without washing or drying. There was always a chance of inhalational ingestion of naphthalene through exposed clothes for all family members, as well as their villagers, who used this practice for years. But, this time for the particular baby, they were added using the mothball tied below the lower end of the shirt with a separate black cloth piece around the waist fastened by a small plastic thread (Figure 1) for the last 10 days. It was especially tied by parents after some local healer’s advice with traditional intent to avoid the effects of so-called bad air or some evil spirit, which was making the baby get frequent ill-health and hospitalization. The mothball was kept continuously near the inguinal region hidden under the shirt or pants, so most likely he had significant dermal absorption causing this severe manifestation, unlike in other family members. On probing the past history of similar illnesses, they revealed the baby had one more hospitalization at the paediatric ward and received a blood transfusion five months back with similar complaints of vomiting, severe anaemia, jaundice, mild fever, hematuria, and hepatomegaly. Being from a sickle endemic zone and belonging to a most susceptible community of central India, at that time sickle cell disease (SCD) versus other hemolytic conditions were thought of, and most workups were done, but as per old medical records and reports retrieved, evidence of hemolysis along with hematuria (but no documented hemoglobinuria) was there, but all workups for SCD, glucose-6-phosphate dehydrogenase (G6PD) deficiency, HUS (Hemolytic Uremic Syndrome), autoimmune disease, and other likely causes of hemolytic anaemia were negative. Thus, after a blood transfusion with other supportive management, he had been discharged with advice to follow up in three months for a repeat G6PD level and other clinical monitoring, but his parents didn’t turn up for proper medical care.

**Figure 2:**
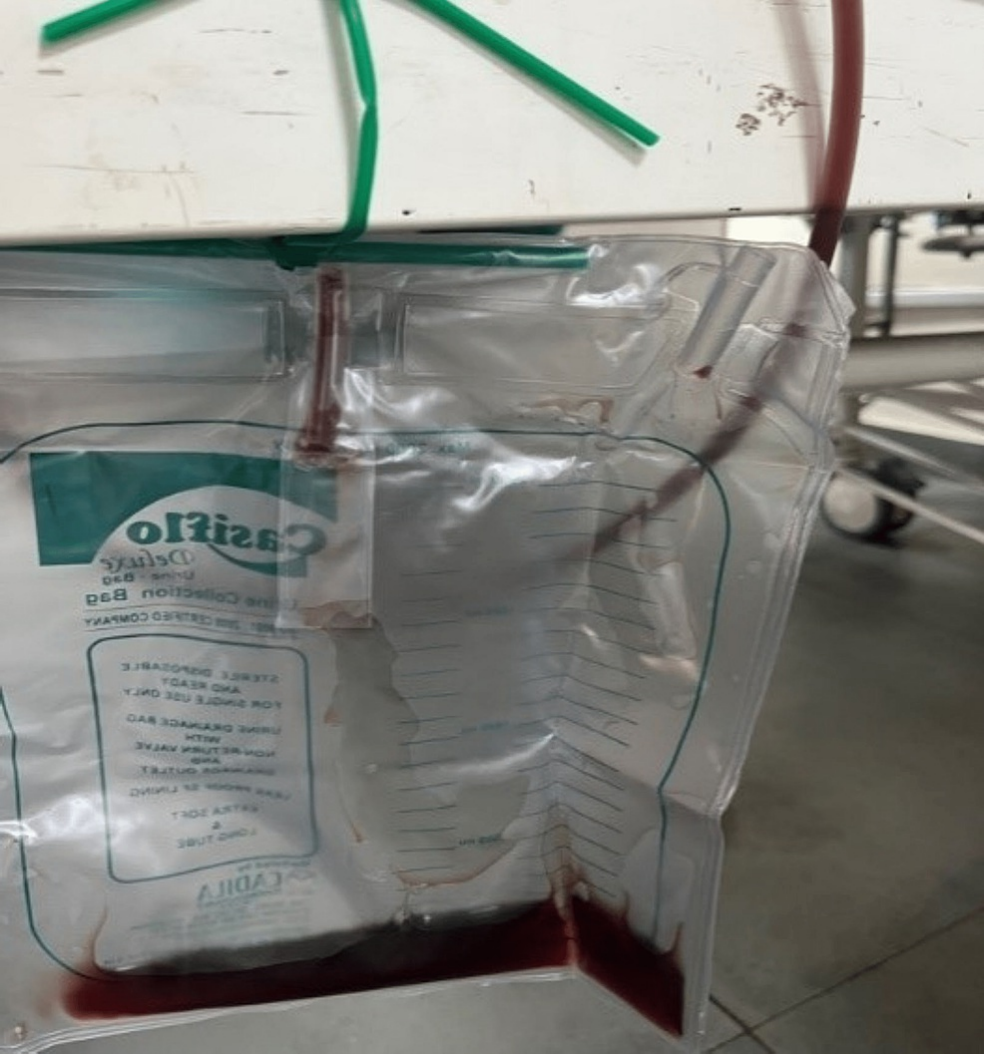
Brown reddish urine on Foley's catheterization

Upon conducting a focused examination (of head-to-toe and relevant systems), no evidence of organomegaly, congestive cardiac failure, or cyanotic congenital heart disease was observed despite severe pallor and hypoxia. Remarkably, no significant abnormalities pertaining to the chest or cardiovascular system were discerned during the clinical assessment. On re-evaluation, in spite of the administration of almost 100% oxygen through NRBM, the oxygen saturation (SpO_2_) exhibited fluctuations (between 60 and 80%) and failed to improve, reaching a maximum level of 85%. The Foley catheterization revealed an adequate amount of urine getting drained since admission, but the colour was reddish brown (as shown in Figure 2). The final triage category assigned was level I, and thus, the child was managed in the red zone bed of the paediatric ED until an ICU bed could be availed.

Diagnostic evaluation

The arterial blood gas (ABG) analysis revealed a pH of 7.5, pCO_2_ of 20.6 mmHg, pO_2_ of 147.5 mmHg, oxygen saturation (SaO_2_) of 88%, bicarbonate (HCO_3_) level of 16.4 mmol/L, and base excess (BE) of 5.2 mEq/L. The elevated arterial oxygen (PaO_2_) levels strongly indicated the presence of methemoglobinemia, which was confirmed by co-oximetry and laboratory testing with a measured methemoglobin (MetHb) level of 23.4%.

Upon overall ED evaluation, the initial clinical impression of hemolytic anaemia was considered, and differential diagnoses kept were SCD and other underlying genetic diseases like G6PD deficiency versus acquired causes like methemoglobinemia, including naphthalene or another oxidative agent poisoning, autoimmune hemolytic anaemia (AIHA), and atypical HUS. Carbon monoxide poisoning and sulfhemoglobinemia could also have been potential differentials for the observed low SpO_2_ levels, but the clinical scenario was not suggestive of them. Given the geographical prevalence of SCD, its presence with associated complications like the acute hemolytic crisis had to be a significant consideration in history, physical examinations, and further laboratory examinations. The child had severe anaemia at ED presentation, as confirmed by a haemoglobin level of 6.2 g/dL, which exhibited a declining trend due to ongoing hemolysis, reaching as low as 4.5 g/dL (see Table 1). Throughout the child's hospital stay, close monitoring of renal and liver functions was conducted, and those showed normal results except for initial values of moderately elevated indirect bilirubin and aspartate transaminase (AST) enzyme. The child's lactate dehydrogenase (LDH) levels were found to be >1200 IU/L, and the corrected reticulocyte count was 4.5%. The urine dipstick revealed 3+ for blood, while the urine routine showed no red blood cells. The urine collection bag showed brownish-red urine every time (as in Figure 2), and hemoglobinuria was also confirmed by laboratory tests of urine, ruling out hematuria and any underlying glomerular aetiology. The Coombs test and anti-nuclear antibody yielded negative results, and the G6PD levels (10.29 U/g of Hb) were within the normal range. High-performance liquid chromatography demonstrated a normal profile of haemoglobins. Thus, ruling out most of the possible differentials, naphthalene-induced oxidative hemolytic anaemia and secondary methemoglobinemia were the final impressions. The oxidative stress produced by naphthalene is known to result in methemoglobinemia-the oxidized form of haemoglobin with ferric iron (Fe3+).

**Table 1:** Serial values of laboratory investigation reports and SpO2 records ED - emergency department, SpO_2_ - oxygen saturation recorded by Pulse-oximeter, AST - aspartate transaminase

Time of Investigation	Investigation variables: Lab report and SpO_2_ value (reference range)				
	Haemoglobin in g/dL (11.5-15.5)	Methemoglobin (< 1.5%)	Spo_2_ (95-100%)	Unconjugated Bilirubin in mg/dL (0.20-0.90)	AST in U/L (12-38)
At the ED presentation (Day 1)	6.2	23.4%	60-80%	4.34	117
After 1^st^ dose of Methylene blue	5.0	21.9%	65-80%	-	-
After 2^nd^ dose of Methylene blue	4.5	21.5%	70-80%	4.75	112
Mid-exchange transfusion	10.7	3.5%	94-96%	-	-
Post-exchange transfusion (Day 2)	10.8	1.2%	98-100%	0.99	47

Therapeutic interventions

The naphthalene ball wrapped around the child's waist was immediately removed in the ED after explaining the risks to the parents. The child received high-flow oxygen, intravenous fluids, and an injection of methylene blue at a dosage of 1 mg/kg, which was repeated twice (after obtaining the previous normal record and the current report of the G6PD level to be normal, i.e., 10.29 U/gramme of Hb), and a high-dose oral ascorbic acid was also started. Despite the diligent application of the prescribed therapeutic interventions, the child exhibited a suboptimal response, as oxygen saturation levels remained persistently low, hovering around the range of 65-70%. Consequently, an exchange transfusion was planned and performed in the ED at a blood volume of 80 mL/kg. During the course of the exchange transfusion, oxygen saturation levels increased, reaching 95% at the mid-transfusion stage and 100% by the end. Additionally, the levels of MetHb exhibited a reducing trend from 23.4% to 21.5%, then measured at 3.5% in the samples taken during the exchange procedure, and further declined to 1.2% following the completion of the exchange transfusion.

Clinical course and follow-up

The initial MetHb levels were significantly higher (i.e., > 20%), and clinically matched the degree of hypoxia as well as symptomatology, which significantly reduced to 1% following the definitive treatments with methylene blue and blood exchange transfusion during the first 48 hours of ED stay only. Table 1 presents the sequential values of haemoglobin, indirect bilirubin levels, AST levels, and MetHb levels during the child's hospitalization.

On the second day of admission, the child exhibited persistent hematuria, which resolved by the third day. Remarkably, following the exchange transfusion, the child experienced a dramatic improvement with the absence of irritability or tachypnoea, and the urine colour returned to its normal colour within the subsequent 72 hours. Furthermore, the bilirubin levels demonstrated a decrease, and no further episodes of hemolysis or hemoglobinuria were observed. At the time of discharge, the methemoglobin level documented was 1.0%.

Additionally, during the three-month follow-up, there were no further instances of similar hemolytic episodes or repeat consumption of moth balls at home. The parents had received comprehensive counselling regarding the detrimental effects of naphthalene balls and were advised on the appropriate storage and careful use of such harmful substances at discharge and follow-up. They were also encouraged to disseminate this knowledge and awareness in their village and community.

## Discussion

When a paediatric patient continues to experience hypoxemia despite receiving 100% supplemental oxygen, the range of potential causes narrows considerably, but the situation becomes challenging. When confronted with clinical scenarios, it is crucial to meticulously evaluate for a range of potential aetiologies like diffusion impairment, ventilation-perfusion (V/Q) mismatch, pulmonary shunting, cyanotic congenital heart diseases, severe anaemia, and high altitude exposure. Given the complexity and potential seriousness of these conditions, it is crucial to consult with a paediatrician and, sometimes, a specialist in pulmonary medicine to conduct a comprehensive clinical and laboratory evaluation.

Naphthalene (C10H8), a bicyclic aromatic hydrocarbon, is poorly soluble in water, and one mothball usually has 0.5-5 g of naphthalene [2]. Although the features of naphthalene toxicity include neurological, gastrointestinal, respiratory, and hepatic effects, the most dreaded complications are severe intravascular hemolysis and acute renal injury secondary to oxidative hemolysis [3-5]. The available literature concerning the toxic dosage of naphthalene is scarce, and its precise threshold remains largely unknown [1]. The toxicity and absorption of naphthalene are heightened when consumed with fatty meals or in the presence of oily skin, leading to its deposition in adipose tissues and subsequent delayed effects. Accidental ingestion by toddlers, mistaking moth balls for candy, accounts for the majority of cases, while literature also reports cases of inhalation and absorption resulting from exposure to clothes stored with mothballs. Due to the varied composition of mothball beyond naphthalene, occasionally including camphor in it, people in multiple places in India use it as cloth deodorant, and some also use it with coconut oil for topical skin application. There are case reports of naphthalene toxicity with such topical dermal application in children. Methemoglobinemia and hemolytic anaemia have both emerged as common findings through multiple case reports on naphthalene toxicity, and this correlation remains true in the index case as well [5,6]. Naphthalene poisoning induces oxidative stress by amplifying the generation of free radicals, leading to lipid peroxidation and subsequent damage to cellular membranes, culminating in cell lysis [7]. The exacerbation of these effects is particularly pronounced in patients with G6PD deficiency. Free radicals also result in the oxidation of the iron (Fe) moiety to the ferric state (Fe 3+), resulting in methemoglobinemia, which doesn’t bind oxygen and also shifts the oxygen dissociation curve to the left [8,9].

The severity of symptoms and the degree of hypoxia or cyanosis are contingent upon the underlying anaemia and the intrinsic mechanism responsible for the clearance of MetHb. Furthermore, these manifestations align with the level of MetHb present, where levels reaching 50% are associated with seizures, dysrhythmia, acidosis, and coma, while levels exceeding 70% are indicative of a fatal outcome. Refractory hypoxia, devoid of any discernible pulmonary or cardiac aetiology, serves as a crucial indicator of methemoglobinemia due to the unique light-absorbing properties of MetHb at varying wavelengths, which are not detected by conventional pulse oximetry. The arterial saturation falsely appears normal in blood gas analysis. The existence of a disparity between the measured SpO_2_ and the actual oxygen-carrying capacity, known as the saturation gap, indicates the presence of methemoglobinemia. Co-oximetry is the confirmatory test, as it gives the total percentage of MetHb in the blood [10]. Further testing of G6PD, haemoglobin M disease, and cytochrome b5R deficiency are needed in selected cases to identify the underlying or additional genetic causes of acute hemolytic presentations [11]. The urine analysis of the current study showed brownish urine with +3 blood, but microscopic examination did not reveal any red blood cells, similar to the findings reported by Giselle Volney et al., which suggested frank hemoglobinuria [8]. Acute kidney injury due to intravascular hemolysis is one of the complications of naphthalene toxicity, as mentioned in the literature, which can be mitigated by meticulous management of fluid balance and urinary alkalization [12,13]. While the reported case presented with respiratory alkalosis, reports of metabolic acidosis have also been documented in the literature [14].

Immediate treatment in ED involves removing the source, stabilizing the airway, providing respiratory and circulatory support, and considering case-specific interventions like fluid administration, inotropes, drug therapy, dialysis, and blood transfusion. Specific treatment modalities include methylene blue, given at a dose of 1 mg/kg IV over 5 minutes when the patient is symptomatic or MetHb is 20-30%. The enzyme NADPH-MetHb reductase reduces methylene blue to leukomethylene blue, which reduces MetHb to haemoglobin. The expected response is seen within 10-60 minutes. Co-oximetry should not be relied upon as a guide to treatment as it cannot differentiate between MetHb and methylene blue. If symptoms persist or recur along with low saturation, a repeat dose of methylene blue can be administered after 30-60 minutes. Methylene blue should be used judiciously in G6PD-deficient individuals as it precipitates hemolysis. Vitamin C can be used in the oral or intravenous form as a supplemental therapy due to its antioxidant properties. High-dose ascorbic acid is another treatment option, but the action takes longer to show therapeutic potential [10]. In refractory cases, hyperbaric oxygen therapy and exchange transfusion or red-cell exchange are recommended; however, the supporting evidence remains limited to a small number of case reports [15-20]. The outcome of the patient is influenced by the quantity of naphthalene exposure and the time lapse before the ED presentation. Not just oral and inhalational routes, but even dermal exposure to naphthalene in children can lead to severe intravascular hemolysis as well as methemoglobinemia, both of which can be effectively treated if promptly diagnosed. To prevent complications such as acute kidney injury, meticulous fluid management, and urinary alkalization play vital roles. The definitive treatment of methemoglobinemia, if indicated, may include methylene blue, ascorbic acid, and exchange transfusions with other supportive care, as needed. We must not forget about the decontamination or removal of the source of exposure in any acute or chronic poisoning to curtail the further risk of recurrent or ongoing toxicity.

## Conclusions

When encountering hypoxia refractory to 100% oxygen and without identifiable cardiac or pulmonary causes, methemoglobinemia should be suspected as a potential underlying condition. This suspicion can be initially raised by pulse oximetry findings and subsequently confirmed through co-oximetry analysis. Similarly, in cases of acute-onset hemolysis, it is crucial to consider the possibility of drug-induced or toxin-mediated aetiologies, emphasizing the importance of actively obtaining a comprehensive but focused history from parents and not forgetting about adequate exposure by practising the ABCDE way of ED assessment. Naphthalene-mediated oxidative stress is responsible for both acute hemolytic anaemia and methemoglobinemia. G6PD deficiency must be ruled out before definitive treatment with methylene blue, as it corrects the methemoglobin level by a complex reduction-oxidation reaction depleting the NADPH reserve, and thus any substance producing oxygen free radicals may produce aggravated toxicity in the milieu of heightened oxidative stress. Especially in rural India, there are certain indigenous and traditional healing practices still prevalent in some parts, where such chemical materials are also used to tie around the waist or neck to get rid of ill-health and evil forces. Although exact dermal or inhalational toxic doses are not known, given the widespread use of naphthalene mothballs in households, it is imperative for paediatricians to know about the varied exposure routes and clinical presentations of naphthalene poisoning.
